# Computational Intelligence Technique for Prediction of Multiple Sclerosis Based on Serum Cytokines

**DOI:** 10.3389/fneur.2019.00781

**Published:** 2019-07-18

**Authors:** Mehendi Goyal, Divya Khanna, Prashant Singh Rana, Timur Khaibullin, Ekaterina Martynova, Albert A. Rizvanov, Svetlana F. Khaiboullina, Manoj Baranwal

**Affiliations:** ^1^Department of Biotechnology, Thapar Institute of Engineering and Technology, Patiala, India; ^2^Department of Computer Science and Engineering, Thapar Institute of Engineering and Technology, Patiala, India; ^3^Republican Clinical Neurological Canter, Republic of Tatarstan, Russian Federation, Kazan, Russia; ^4^Institute of Fundamental Medicine and Biology, Kazan Federal University, Kazan, Russia; ^5^Department of Microbiology and Immunology, University of Nevada, Reno, NV, United States

**Keywords:** multiple sclerosis, cytokines, serum, machine learning, prediction

## Abstract

Multiple sclerosis (MS) is a neurodegenerative disease characterized by lesions in the central nervous system (CNS). Inflammation and demyelination are the leading causes of neuronal death and brain lesions formation. The immune reactivity is believed to be essential in the neuronal damage in MS. Cytokines play important role in differentiation of Th cells and recruitment of auto-reactive B and T lymphocytes that leads to neuron demyelination and death. Several cytokines have been found to be linked with MS pathogenesis. In the present study, serum level of eight cytokines (IL-1β, IL-2, IL-4, IL-8, IL-10, IL-13, IFN-γ, and TNF-α) was analyzed in USA and Russian MS to identify predictors for the disease. Further, the model was extended to classify MS into remitting and non-remitting by including age, gender, disease duration, Expanded Disability Status Scale (EDSS) and Multiple Sclerosis Severity Score (MSSS) into the cytokines datasets in Russian cohorts. The individual serum cytokines data for the USA cohort was generated by Z score percentile method using R studio, while serum cytokines of the Russian cohort were analyzed using multiplex immunoassay. Datasets were divided into training (70%) and testing (30%). These datasets were used as an input into four machine learning models (support vector machine, decision tree, random forest, and neural networks) available in R programming language. Random forest model was identified as the best model for diagnosis of MS as it performed remarkable on all the considered criteria i.e., Gini, accuracy, specificity, AUC, and sensitivity. RF model also performed best in predicting remitting and non-remitting MS. The present study suggests that the concentration of serum cytokines could be used as prognostic markers for the prediction of MS.

## Introduction

Multiple sclerosis (MS) is a chronic disease of the central nervous system (CNS) caused by chronic inflammation and autoimmune response. MS can be classified on the basis of onset of symptoms and their progression into relapsing remitting (symptoms appearing and disappearing), primary progressive (progressive symptom elevation), and secondary progressive (relapse-remitting MS development to progressive MS) multiple sclerosis. The disease is characterized by demyelinating areas in the brain and spinal cord which appear as plaques or lesions in the white and gray matter (1, 2). Blood Brain Barrier (BBB) was shown to be affected, which explains the presence of circulating leukocytes into the brain matter (3). The auto-reactive T lymphocytes penetrating BBB could target neuroglia leading to more damage within the brain and thus exposing myelin antigens. These auto-reactive T cells can cause deterioration of the myelin sheath, which is essential for signal transmission within the brain (4). Depending on the varied locations of lesions in brain, clinical symptoms of MS may vary including vision loss, numbness, fatigue, movement difficulties, and many more (5).

Neuronal damage and neuroglial activation could cause the secretion of various cytokines which are involved in differentiation of Th1, Th2, Th9, and Th17 lymphocytes (6). Studies have shown changes in various cytokines level in serum and cerebrospinal fluid (CSF) of MS patients as compared to controls (7–9). These cytokines are associated with Th1 (IFN-γ, TNF-α, IL-2) and Th2 (IL-4, IL-5, IL-13, IL-6) type immune responses. Also, activation of Th17 and Th9, secreting IL-17 and IL-9, respectively, was shown to play role in the progression of MS (10). Interestingly, loss of the natural regulatory T cells (T_reg_) function was demonstrated as one of the factors leading to MS (11, 12). It is believed that suppression of the T_reg_ population can lead to proliferation of auto-reactive T cells in MS (11).

The analysis of body fluids such as blood, saliva, cerebrospinal fluid, and urine is often used to diagnose various diseases at the early stage. This analysis can be highly accurate and cost effective than the conventional diagnostic techniques such as computed tomography (CT), magnetic resonance imaging (MRI) scans, and tissue biopsies. The body fluids are commonly analyzed to determine changes in biomolecules which are either directly or indirectly associated with the disease progression. Since, blood cytokines is known to be affected in MS, hence we propose that changes in cytokine could be used as a prognostic markers for MS diagnosis.

Machine learning approaches were successfully employed for prediction of Alzheimer's disease, diabetes, inflammatory bowel disease, and diagnosis of glaucoma (13–16). Recently, machine learning approach was applied into demographic dataset to predict MS disease course (17). Martins et al. analyzed thirteen inflammatory cytokines in 833 MS patients and 117 controls of USA population (18). Eight out of thirteen cytokines were found to differ significantly in MS as compared to controls (18). These eight cytokines were also analyzed in MS patients and controls of Russian cohort. In current study, four machine learning models were applied to predict MS using these eight cytokines (IL-1β, IL-2, IL-4, IL-8, IL-10, IL-13, IFN-γ, and TNF-α) data of USA and Russian cohorts. Further, machine learning models were also used to classify MS into remitting and non-remitting based on eight cytokine serum level, age, gender, disease duration, Expanded Disability Status Scale (EDSS) and Multiple Sclerosis Severity Score (MSSS).

## Materials and Methods

**Figure 1:**
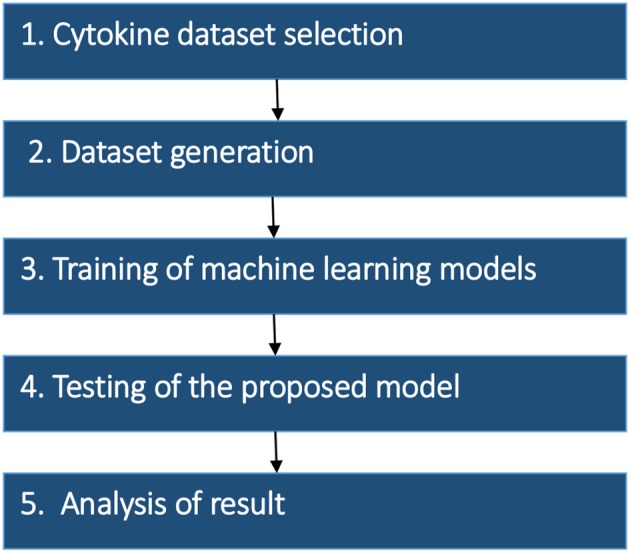
Methodology of the proposed work.

The research strategy of the proposed model was divided into the five stages: (1) Dataset selection, (2) Dataset generation, (3) Training of machine learning models (4) Testing of the proposed model, and (5) Analysis of the result. The methodology of proposed work and details of each stage are summarized in Figure 1.

### Dataset Selection

Concentration data of eight cytokines (IL-1β, IL-2, IL-4, IL-8, IL-10, IL-13, IFN-γ, and TNF-α) in serum of MS patients and controls was selected from two different studies of USA and Russian population. Out of the two independent USA studies, one analyzed the concentration of serum cytokines in 833 MS patients and 117 healthy volunteers using multiplex immunoassay (18) while the other group analyzed the concentrations of serum cytokines in 26 MS patients and 11 controls (19). Data on eight serum cytokines (IL-1β, IL-2, IL-4, IL-8, IL-10, IL-13, IFN-γ, and TNF-α) in 97 MS patients and 71 controls in Russian cohort was also included into the analysis. There were 53 females and 18 males average age 28.6 ± 8.8 years, in Russian control cohort. The demographic and clinical features of 97 Russian MS patients are summarized in Table 1.

**Table 1:** Demographic and clinical details of MS patients from Russian cohort.

**Characteristic**		**Number or mean ± SD**
Age		39.1 ± 13.4
Gender	Female	67
	Male	30
MS types	Relapsing remitting	46
	Secondary progressive	31
	Primary progressive	20
Disease duration		3.9 ± 2.2
EDSS		2.6 ± 1.5
MSSS		4.9 ± 2.3
Patients on treatment		22
Not on treatment		75

### Dataset Generation

Dataset containing USA populations was generated using Z score percentile based method while Russian cytokine data was analyzed using multiplex magnetic bead-based antibody detection assays.

### Z Score Percentile Method

Cytokine data from two previously published USA studies was reported in the mean ± standard deviation (SD)/standard error of mean (SEM) format. To convert SEM into SD, the SEM was multiplied by square root of total number (n). One of the major challenges was to generate the individual cytokines data from reported values as the data was mostly available as mean ± SD/SEM. Data was generated by two methods: solving the series of non-linear equations and Z score percentile based approach. To choose best method for data analysis, random values of 50 cytokines were taken, and the actual values were compared with the generated values from Z score method and non-linear systems equations (data not shown). The data generated by Z score method was found to be more accurate. Hence, to generate the raw data from mean ± SD/SEM, Z score percentile method was used, where the population was presumed to follow the normal distribution (20). The Z score percentile method was implemented in R (an open source software licensed under GNU GPL) to calculate individual data. In this method, 99.7% of the total population was included and the remaining 0.3% was considered outliers and was excluded from the analysis (Supplementary Figure 1).

### Cytokine Analysis

Ninety seven MS patients, admitted to the Republican Clinical Neurological Center, Republic of Tatarstan, Russian Federation were recruited into the study. MS diagnosis was based upon clinical presentation and brain MRI results. Serum samples were collected from each patient and control. Informed consent was obtained from each subject according to the clinical and experimental research protocol, approved by the Biomedicine Ethic Expert Committee of Republican Clinical Neurological Center, Republic of Tatarstan, Russian Federation (No.218; 11.15.2012).

Serum cytokines (IL-1β, IL-2, IL-4, IL-8, IL-10, IL-13, IFN-γ, and TNF-α) were analyzed using Pro Human Cytokine 27-plex Bio-Plex (Bio-Rad, Hercules, CA, USA) multiplex magnetic bead-based antibody detection kits following the manufacturer's instructions. Serum aliquots (50 μl) were used for the analysis with a minimum of 50 beads per analyte acquired. Median fluorescence intensities were measured using a Luminex 200 analyzer. Data collected was analyzed with MasterPlex CT control software and MasterPlex QT analysis software (Hitachi Software, San Bruno, CA, USA). Standard curve for each analyte was generated using standards provided by the manufacturer.

### Machine Learning Methods

Four machine learning models, Random Forest (RF) (21), Decision Tree (DT), Support Vector Machine (SVM) (22), and Neural Network (NN) (23) were used in the study. The required packages and tuning parameters to obtain the optimum results using these models are summarized in Table 2. The models were trained based on equation which includes factors required to predict the target 1 (MS vs. control) or classify target 2 (remitting vs. non-remitting MS).

$$\begin{array}{l} Target\;1.1\sim f(IL1\beta +IL2+IL4+IL8+IL10+IL13\\ \;+IFN\gamma +TNF\alpha )\;\\ Target\;1.2\sim f(IL1\beta +IL2+\;IL4+\;IL8+\;IL10\\ \;+IL13+\;IFN\gamma \;+TNF\alpha +Age+Gender)\\ \;Target\;2\sim f(IL1\beta +IL2+\;IL4+\;IL8+\;IL10\\ \;+IL13+\;IFN\gamma ,\;+TNF\alpha +Age+Gender\\ \;+EDSS+MSSS+Disease\;duration\text{)} \end{array}$$

**Table 2:** Tuning parameters of machine learning models.

**Model**	**Method**	**Required package**	**Tuning parameter**
SVM	Ksvm	Kernlab	Kernel radial basis
DT	Rpart	rpart	Min split = 20, Max depth = 30
RF	Rf	Random forest	mtry = 2, number of tree = 500
NN	nn.train	Deepnet	hidden layer = 5

*SVM, Support vector machine; DT, Decision tree; RF, Random forest; NN, Neural network*.

### Model Evaluation

The performance of models was evaluated using various parameters such as Gini, AUC, accuracy, specificity, and sensitivity (24). The following equations were used to calculate these parameters:

$$\begin{array}{l} \;Gini=2\;\times AUC-1\\ Accuracy=\;\frac{TP+TN}{TP+TN+FN+FP}\times 100\\ Sensitivity=\frac{TP}{TP+FN}\\ Specificity=\frac{TN}{TN+FP} \end{array}$$

Where,

TN: True negative; TP: True positive; FP: False positive; FN: False negative. AUC: AUC (Area under Curve) is area under Receiver Operating Characteristics (ROC) curve which is calculated to measure the quality of model. Higher AUC value depicts a good quality model.

### Repeated K-Fold Cross Validation

K-fold cross validation was done to test the robustness of proposed model by increasing the number of runs in model. In this method, K-folds are repeated n times to trace out the fluctuations in the model accuracy. If low variation in accuracy is identified, the model is identified as robust and the predictions to be reliable. In the present study, the dataset was divided into six equal portions and 6-fold cross validation was repeated three times to avoid discrepancies.

## Results

### The Proposed Predictive Model

**Figure 2:**
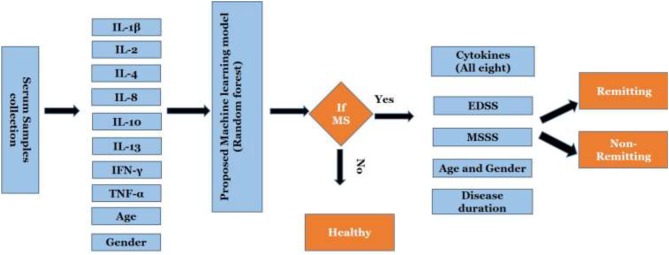
Work flow of the proposed scheme. EDSS, Expanded Disability Status Scale; MSSS, Multiple Sclerosis Severity Score.

The proposed algorithm to predict and classify MS is summarized in Figure 2. The model is based on eight cytokines level in serum for MS and control. Datasets of cytokine levels, age and gender were used as input for machine learning model to predict if a person is having MS or not. Once MS is diagnosed, the model will be able to classify MS into remitting and non-remitting MS based on serum cytokines, age, gender, disease duration, EDSS, and MSSS.

Four machine learning models were employed to predict MS using dataset including 910 MS patients and 199 controls. The dataset was prepared by random shuffling of USA and Russian cohorts and then the data was divided into training (70%) and testing (30%) subsets. The data was divided as follows: 900 (training dataset) and 209 (testing dataset). The training dataset consisted of unbalanced data on MS patients (750) and controls (150) which was further distributed by dividing patient data into five subsets to create a balance between the patient and control datasets. All four machine learning models were trained separately using each balanced dataset. All five trained models were then tested by using test dataset. Predictions generated via five trained models were combined using majority voting ensemble technique. Using SVM, DT, and RF, fare accuracy of MS prediction was demonstrated (83–91%). When additional parameters used for the analysis (Gini, AUC, specificity, and sensitivity) were looked, RF model demonstrated the best performance as compared to other models. Therefore, RF was selected as model for the prediction of MS and used for validation (Table 3).

**Table 3:** Performance of machine learning models based on evaluation parameters.

**Model name**	**Gini**	**Accuracy**	**AUC**	**Sensitivity**	**Specificity**
SVM	0.862	87.56	0.931	0.5	0.633
DT	0.715	83.73	0.858	0.069	0.541
**RF**	**0.914**	**90.91**	**0.957**	**0.756**	**0.857**
NN	0.566	45.45	0.783	0.456	0.082

*SVM, Support vector machine; DT, Decision tree; RF, Random forest; NN, Neural network. The bold values suggests that Random Forest (RF) was selected as the best predictive model based on the listed evaluation parameters*.

**Figure 3:**
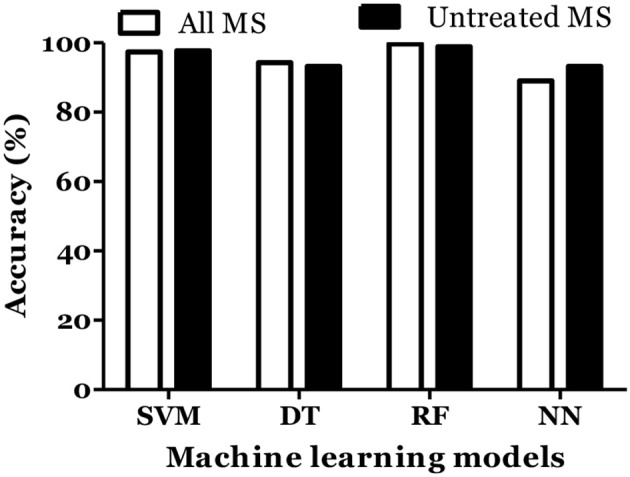
Prediction accuracy of different models to diagnose MS in Russian cohort. All MS: MS patients which includes the patients undergoing and not on treatment when serum samples was collected. Untreated MS: MS patients which includes the patients which were not on treatment when serum samples was collected.

**Figure 4:**
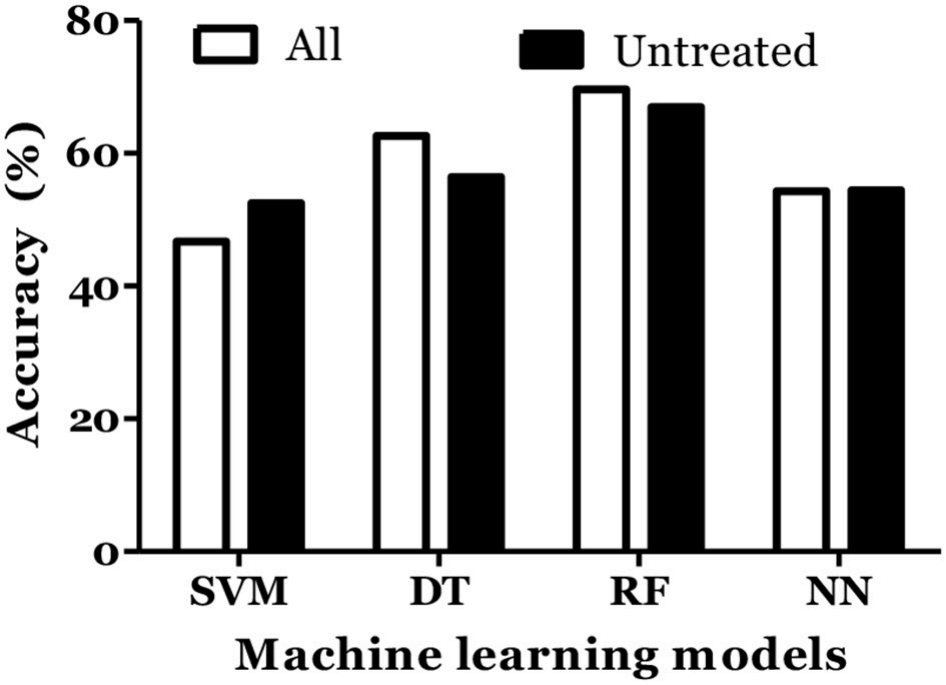
Prediction accuracy of different models to classify MS types in Russian cohort. All MS: MS patients which includes the patients undergoing treatment and not on treatment when serum samples was collected. Untreated MS: MS patients which includes the patients which were not on treatment when serum samples was collected.

The prediction of MS was also done with inclusion of age and gender along with cytokine values in Russian cohort where datasets were divided into training (70%) and testing (30%). The accuracy of MS diagnosis for different models was within the range of 89–99% (Figure 3). RF model demonstrated 70% accuracy in classifying remitting and non-remitting MS while the percentage accuracy for DT, NN, and SVM models was 63, 54, and 47, respectively (Figure 4). In the Russian MS cohort, 97 patients, consisting of 22 patients taking medication, were included. Therefore, to compare the effect of MS treatment on MS prediction accuracy, 97 MS patients were compared with 75 MS patients without treatment. Data analysis did not reveal difference between these two groups (Figures 3, 4). Thus, it was concluded that, the MS prediction accuracy is not affected by inclusion of patients undergoing treatment.

**Figure 5:**
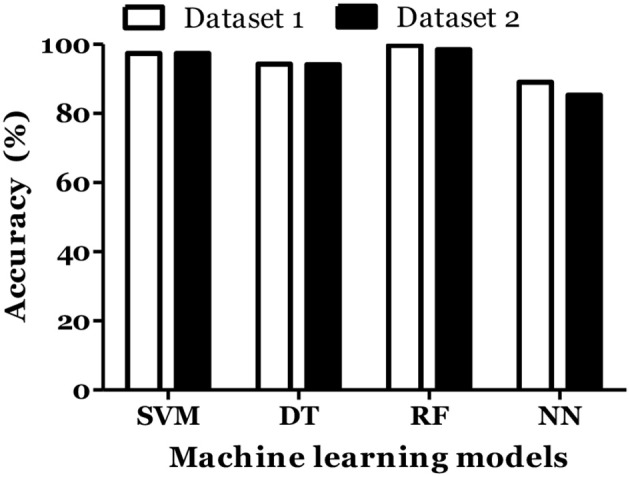
Prediction accuracy of different models of different datasets to diagnose MS in Russian cohort. Dataset 1: IL1-β + IL-2 + IL-4 + IL-8 + IL-10 + IL-13 + IFN-γ + TNF-α + Age + Gender. Dataset 2: IL1-β + IL-2 + IL-4 + IL-6 + IL-8 + IL-10 + IL-13 + IFN-α + IFN-γ + TNF-α + Age + Gender.

**Figure 6:**
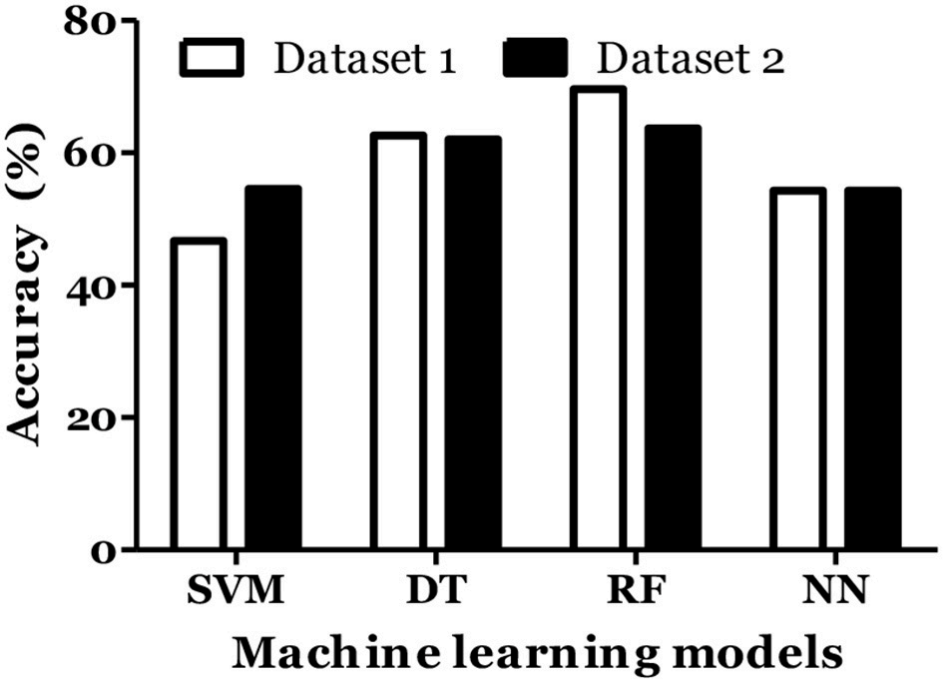
Prediction accuracy of different models of different datasets to classify MS types in Russian cohort. Dataset 1: IL1-β + IL-2 + IL-4 + IL-8 + IL-10 + IL-13 + IFN-γ + TNF-α + Age + Gender + Disease duration + EDSS + MSSS. Dataset 2: IL1-β + IL-2 + IL-4 + IL-6 + IL-8 + IL-10 + IL-13 + IFN-α + IFN-γ + TNF-α + Age + Gender + Disease duration + EDSS + MSSS.

IL-6 and IFN-α were shown to play role in MS pathogenesis (25, 26). Therefore, we included these cytokines in dataset and calculated the MS prediction accuracy. We have found that inclusion of these cytokines did not improve the accuracy of MS prediction and classification (Figures 5, 6).

### Validation of the Proposed Model

**Figure 7:**
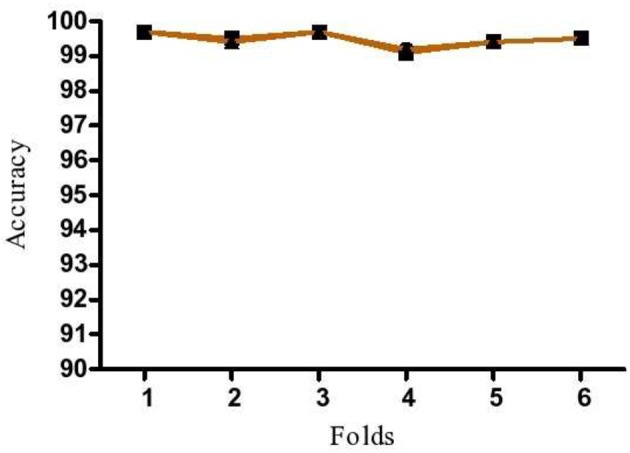
Repeated K-fold validation of the proposed model. Data is mean of three independent runs.

**Figure 8:**
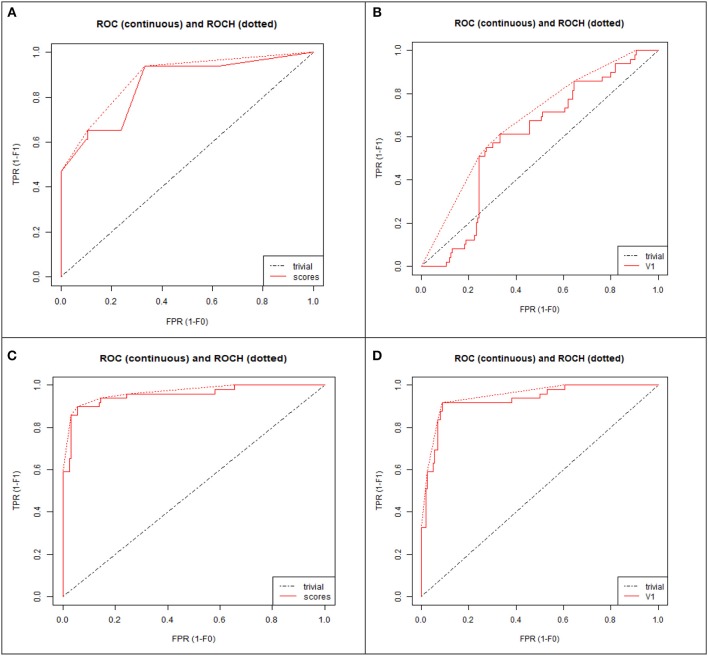
ROC curve plot of four models: **(A)** Decision tree (DT), **(B)** Neural network (NN), **(C)** Random forest (RF), **(D)** Support vector machine (SVM). TPR, True positive rate; FPR, False positive rate.

To demonstrate that the trained model is not overfitted, underfitted or biased, repeated 6-fold cross validation was performed. The accuracy of the proposed model was evaluated by repeated K-fold cross validation (Figure 7). The Receiver operating Characteristic (ROC) is the representation of the true positive rate (sensitivity) and false positive rate (1 specificity) of the models where for each data point, the sensitivity and specificity are calculated to plot the graph. The area under the curve (AUC) can be considered as the criterion for the measurement of the discriminative ability of the model to distinguish well-among the patients and controls. Receiver operating Characteristic (ROC) curve plots for each model were generated to demonstrate the performance of each model (Figure 8). It was observed that the RF model is performing well as compared with other models (Figure 8).

## Discussions

The pathogenesis of MS is complex and involves multiple factors which makes prediction and early diagnosis of the disease challenging. Recently, different computational methods were applied to develop interactive design and optimisation of the synthetic biological system to study pathogenesis of diabetes (27). This study was designed to develop novel approaches for diagnosis of the disease; because, early diagnosis of the disease could significantly increase the success rate of the current treatment. Artificial intelligence holds a great potential for early diagnosis and prediction of the treatment outcome. Several machine learning models have been developed to predict development of the heart diseases, Parkinson's disease and breast cancers (28–30). In this study, RF model was identified as the best to predict MS based on eight cytokine levels in serum. RF model has also shown good accuracy in classifying MS into remitting and non-remitting.

MS is a neurological disease highly prevalent in many European countries, USA, Canada and Australia (31). Clinically, MS is characterized by neurological dysfunction which often leads to a disability (32). Despite the advances made in our understanding of MS pathogenesis, prognostic markers for prediction of the disease remain largely unknown. Cytokines were shown to be consistently affected in serum of MS (18). Also, multiple studies have demonstrated that cytokines play a crucial role in the pathogenesis of MS (33, 34). For example, Martins et al have shown that seven cytokines (IL-2, IL-4, IL-10, IL-13, IL1β, IFN-γ, and TNF-α) were significantly elevated in MS patients while IL-8 was significantly lower in MS as compared to controls (18). Interestingly, IL-2, IL-4, IL-10, IL-13, IL1β, IFN-γ, and TNF-α serum level was found elevated in Russian MS as compared to controls, which was similar to that found in USA cohort. These data suggest that the pathogenesis of MS in Russian and USA could be similar. The only exception was changes in serum level of IL8, which was lower in USA and higher in Russian MS as compared to the respective controls. IL-8 is polypotent cytokine involved in regulation of inflammation, recruiting neutrophils, basophils, T lymphocytes, NK cells as well as enhancing the permeability of endothelial barrier (35–38). Difference in IL-8 serum level in Russian and USA MS cohort could reflect the dissimilarities in the disease pathogenesis which could be related to the genetic predisposition, sun exposure, vitamin D production, smoking, etc.

We suggest that changes in serum cytokine levels could be used as predictors or diagnostic biomarkers for MS. Data on serum cytokine level in USA MS cohort was used in our study to develop the machine learning model. To increase the number of samples, data from another report on USA MS serum cytokine levels was included into the analysis (19). The raw data from these two studies was calculated via Z score percentile method. In the resulting synthetic data, the real experimental data obtained by multiplex immunoassay from Russian cohort was included to have high quality prediction. Four machine learning models were trained to predict MS where prediction was based on combined effect of level of eight cytokines in serum. Three models (SVM, DT, and RF) showed good accuracy for MS prediction. The model performance was further evaluated using additional factors (Gini, AUC, specificity and sensitivity). RF model has shown the best performance in each evaluation parameters. This data suggest that RF analysis of eight cytokine (IL-1β, IL-2, IL-4, IL-8, IL-10, IL-13, IFN-γ, and TNF-α) levels in serum could be used to predict MS. RF model has shown the accuracy of 70% to classify MS into remitting vs. non-remitting where age, gender, disease duration, EDSS, and MSSS in addition to cytokines levels were included as classification parameters. This data corroborates previous report where the accuracy of MS disease course was 60–70% when demographic (age, disease onset, gender, and smoking history) and clinical factors (expanded disability status scale, visual disability score, and mental disability score) were included into the prediction model (17).

IL-6 and IFN-α are the inflammatory cytokines which also affected in MS (25, 26). Therefore, prediction and classification of MS algorithm was designed including these cytokines. Interestingly, adding IL-6 and INF-α did not improve the accuracy of MS diagnosis and classification. This suggests that although IL-6 and INF-α contribute into MS pathogenesis, data on level of eight cytokines (IL-1β, IL-2, IL-4, IL-8, IL-10, IL-13, IFN-γ, and TNF-α) in serum provides sufficient input data to diagnose and classify MS.

Analysis of Cerebrospinal fluid (CSF) demonstrated association between cytokines and MS pathogenesis; however, data remains inconsistent (39). In our previous report, ten (IL-2RA, CCL5, CCL11, CXCL1, CXCL10, CXCL12, MIF, IFN-γ, TRAIL, and SCF) out of forty eight cytokines were found elevated in MS as compared to non-MS controls (40). IFN-γ level was only found to be increased in CSF of MS in this study, while the remaining cytokines (IL-1β, IL-2, IL-4, IL-8, IL-10, IL-13, IFN-γ, and TNF-α), used in our prediction model, did not change significantly as compared to controls. Therefore, we did not include CSF cytokine data into our prediction model. Additionally, CSF collection painful and invasive procedure requiring highly trained personnel. Also, CSF analysis is not always required for MS diagnosis. In contrast, MS serum samples are often collected for routine clinical analysis, making them readily available for cytokine detection. Current approach could also be applied to differentiate MS from other neuro-inflammatory diseases.

## Conclusion

Early diagnosis of MS remains a challenge since the disease develops slowly and clinical symptoms are often identified when brain tissue is already damaged. In the present study, RF model was found to have an accuracy of 91% which suggests that it could be applied to predict MS using serum level of eight cytokines (IL-1β, IL-2, IL-4, IL-8, IL-10, IL-13, IFN-γ, and TNF-α). Further, the accuracy of MS classification into remitting vs. non-remitting was observed to 70% by RF with inclusion of age, gender, diseases duration, EDSS and MSSS in addition to serum cytokines. This is the first study where eight cytokine levels in serum was used to predict MS in two distinct cohorts of patients.

## Ethics Statement

Informed consent was obtained from each subject according to the clinical and experimental research protocol, approved by the Biomedicine Ethic Expert Committee of Republican Clinical Neurological Center, Republic of Tatarstan, Russian Federation (No.218; 11.15.2012).

## Author Contributions

MG: original idea generation, literature reviews for MS cytokines data, computational work that includes generation of USA MS cytokines data, compilation of results and figures, manuscript writing. DK: running of machine learning models and results generation of the models. PR: supervised the research work of machine learning models. TK and EM: involved in collection of MS and control samples and MS clinical data. SK: cytokines analysis of Russian population and manuscript editing. AR: arranging the work of Russian cytokines data analysis that includes the MS and control samples. MB: formulation of idea, overall responsible for coordinating the research project and managing multisite collaboration, writing the manuscript.

### Conflict of Interest Statement

The authors declare that the research was conducted in the absence of any commercial or financial relationships that could be construed as a potential conflict of interest.

## References

[1] Ruuls SR Sedgwick JD. Cytokine-directed therapies in multiple sclerosis and experimental autoimmune encephalomyelitis. Immunol Cell Biol. (1998) 76:65–73. 10.1046/j.1440-1711.1998.00715.x 9553778

[2] Kantarci OH. A new dawn for genetic association studies in multiple sclerosis. Neurol Genet. (2016) 2:e93. 10.1212/NXG.0000000000000093 27540593 PMC4974844

[3] Dendrou CA Fugger L Friese MA. Immunopathology of multiple sclerosis. Nat Rev Immunol. (2015) 15:545–8. 10.1038/nri3871 26250739

[4] Goverman J. Autoimmune T cell responses in the central nervous system. Nat Rev Immunol. (2009) 9:393. 10.1038/nri2550 19444307 PMC2813731

[5] Vellinga M Geurts J Rostrup E Uitdehaag B Polman C Barkhof F. Clinical correlations of brain lesion distribution in multiple sclerosis. J Mag Reson Imaging. (2009) 29:768–3. 10.1002/jmri.21679 19306365

[6] Trenova A Slavov G Cytokines in multiple sclerosis–possible targets for immune therapies. J Neurol Exp Neurosci. (2016) 1:25–9. 10.17756/jnen.2016-006

[7] Kallaur AP Oliveira SR Delicato de Almeida ER Kaminami Morimoto H Lopes J de Carvalho Jennings Pereira WL Cytokine profile in relapsing remitting multiple sclerosis patients and the association between progression and activity of the disease. Mol Med Rep. (2013) 7:1010–20. 10.3892/mmr.2013.1256 23292766

[8] Taşdemir N Karaca EE Ece A Yücel Y Dikici S Taşdemir MS Multiple sclerosis: relationships between cytokines, MRI lesion burden, visual evoked potentials and disability scores. Eur J Gen Med. (2010) 7:167–73. 10.29333/ejgm/82845

[9] Khaiboullina SF Gumerova AR Khafizova IF Martynova EV Lombardi VC Bellusci S. CCL27: novel cytokine with potential role in pathogenesis of multiple sclerosis. BioMed Res Int. (2015) 2015:189638. 10.1155/2015/189638 26295034 PMC4532821

[10] Amedei A Prisco D D'elios MM. Multiple sclerosis: the role of cytokines in pathogenesis and in therapies. Int J Mol Sci. (2012) 13:13438–60. 10.3390/ijms131013438 23202961 PMC3497335

[11] Kumar M Putzki N Limmroth V Remus R Lindemann M Knop D. CD4+ CD25+ FoxP3+ T lymphocytes fail to suppress myelin basic protein-induced proliferation in patients with multiple sclerosis. J Neuroimmunol. (2006) 180:178–84. 10.1016/j.jneuroim.2006.08.003 17011048

[12] Putzki N Kumar M Kreuzfelder E Grosse-Wilde H Diener H Limmroth V Mitoxantrone does not restore the impaired suppressive function of natural regulatory T cells in patients suffering from multiple sclerosis. Eur Neurol. (2009) 61:27–32. 10.1159/000165346 18948697

[13] Mossotto E Ashton J Coelho T Beattie R MacArthur B Ennis S. Classification of paediatric inflammatory bowel disease using machine learning. Sci Rep. (2017) 7:2427. 10.1038/s41598-017-02606-2 28546534 PMC5445076

[14] Dagliati A Marini S Sacchi L Cogni G Teliti M Tibollo V. Machine learning methods to predict diabetes complications. J Diabetes Sci Technol. (2017) 12:295–302. 10.1177/1932296817706375 28494618 PMC5851210

[15] Abós A Baggio HC Segura B García-Díaz AI Compta Y Martí MJ. Discriminating cognitive status in Parkinson's disease through functional connectomics and machine learning. Sci Rep. (2017) 7:45347. 10.1038/srep45347 28349948 PMC5368610

[16] Kim SJ Cho KJ Oh S. Development of machine learning models for diagnosis of glaucoma. PLoS ONE. (2017) 12:e0177726. 10.1371/journal.pone.0177726 28542342 PMC5441603

[17] Zhao Y Healy BC Rotstein D Guttmann CR Bakshi R Weiner HL. Exploration of machine learning techniques in predicting multiple sclerosis disease course. PLoS ONE. (2017) 12:e0174866. 10.1371/journal.pone.0174866 28379999 PMC5381810

[18] Martins TB Rose JW Jaskowski TD Wilson AR Husebye D Seraj HS. Analysis of proinflammatory and anti-inflammatory cytokine serum concentrations in patients with multiple sclerosis by using a multiplexed immunoassay. Am J Clin Pathol. (2011) 136:696–704. 10.1309/AJCP7UBK8IBVMVNR 22031307

[19] Cala CM Moseley CE Steele C Dowdy SM Cutter GR Ness JM. T cell cytokine signatures: biomarkers in pediatric multiple sclerosis. J Neuroimmunol. (2016) 297:1–8. 10.1016/j.jneuroim.2016.04.015 27397070 PMC4940981

[20] Wang Y Chen HJ Use of percentiles and z-scores in anthropometry. In: Preedy VR, editor. Handbook of Anthropometry. New York, NY: Springer (2012). p. 29–48. 10.1007/978-1-4419-1788-1_2

[21] RColorBrewer S Liaw A Wiener M Liaw MA Package ‘randomForest’ (2015). Available online at: https://cran.r-project.org/web/packages/randomForest/randomForest.pdf

[22] Karatzoglou A Smola A Hornik K Karatzoglou MA Package ‘kernlab’ (2016). Available online at: http://cran.rediris.es/web/packages/kernlab/kernlab.pdf

[23] Rong X Deepnet: Deep Learning Toolkit in R. R Package Version 0.2. (2014). Available online at: http://CRAN.R-project.org/package=deepnet

[24] Khanna D Rana PS. Multilevel ensemble model for prediction of IgA and IgG antibodies. Immunol Lett. (2017) 184:51–60. 10.1016/j.imlet.2017.01.017 28214535

[25] Göbel K Ruck T Meuth SG. Cytokine signaling in multiple sclerosis: lost in translation. Mult Scler J. (2018) 24:432–9. 10.1177/1352458518763094 29512406

[26] Reder AT Feng X. How type I interferons work in multiple sclerosis and other diseases: some unexpected mechanisms. J Interferon Cytokine Res. (2014) 34:589–99. 10.1089/jir.2013.0158 25084175 PMC4118715

[27] Miller M Hafner M Sontag E Davidsohn N Subramanian S Purnick PE. Modular design of artificial tissue homeostasis: robust control through synthetic cellular heterogeneity. PLoS Comput Biol. (2012) 8:e1002579. 10.1371/journal.pcbi.1002579 22829755 PMC3400602

[28] Nikhar S Karandikar A Prediction of heart disease using machine learning algorithms. Int J Eng Technol. (2018) 7:363–6. 10.14419/ijet.v7i2.32.15714

[29] Bind S Tiwari AK Sahani AK A survey of machine learning based approaches for Parkinson disease prediction. Int J Comp Sci Inform Technol. (2015) 6:1648–55.

[30] Ahmad L Eshlaghy A Poorebrahimi A Ebrahimi M Razavi A Using three machine learning techniques for predicting breast cancer recurrence. J Health Med Inform. (2013) 4:124 10.4172/2157-7420.1000124

[31] Leray E Moreau T Fromont A Edan G. Epidemiology of multiple sclerosis. Rev Neurol. (2016) 172:3–13. 10.1016/j.neurol.2015.10.006 26718593

[32] Frohman EM Racke MK Raine CS. Multiple sclerosis—the plaque and its pathogenesis. New Engl J Med. (2006) 354:942–55. 10.1056/NEJMra052130 16510748

[33] Becher B Spath S Goverman J. Cytokine networks in neuroinflammation. Nat Rev Immunol. (2017) 17:49–59. 10.1038/nri.2016.123 27916979

[34] Codarri L Fontana A Becher B. Cytokine networks in multiple sclerosis: lost in translation. Curr Opin Neurol. (2010) 23:205–11. 10.1097/WCO.0b013e3283391feb 20442570

[35] Fogh K Larsen C Iversen L Kragballe K. Interleukin-8 stimulates the formation of 15-hydroxy-eicosatetraenoic acid by human neutrophils *in vitro*. Agents Actions. (1992) 35:227–31. 10.1007/BF01997504 1529797

[36] Nourshargh S Perkins J Showell H Matsushima K Williams T Collins P. A comparative study of the neutrophil stimulatory activity *in vitro* and pro-inflammatory properties *in vivo* of 72 amino acid and 77 amino acid IL-8. J Immunol. (1992) 148:106–11. 1727857

[37] Baggiolini M Dewald B Moser B lnterleukin-8 and related chemotactic cytokines—CXC and CC chemokines. Adv Immunol. (1993) 55:97–179. 10.1016/S0065-2776(08)60509-X 8304236

[38] Mukaida N Harada A Matsushima K. Interleukin-8 (IL-8) and monocyte chemotactic and activating factor (MCAF/MCP-1), chemokines essentially involved in inflammatory and immune reactions. Cytokine Growth F R. (1998) 9:9–23. 10.1016/S1359-6101(97)00022-1 9720753

[39] Kothur K Wienholt L Brilot F Dale RC. CSF cytokines/chemokines as biomarkers in neuroinflammatory CNS disorders: a systematic review. Cytokine. (2016) 77:227–37. 10.1016/j.cyto.2015.10.001 26463515

[40] Khaibullin T Ivanova V Martynova E Cherepnev G Khabirov F Granatov E. Elevated levels of proinflammatory cytokines in cerebrospinal fluid of multiple sclerosis patients. Front immunol. (2017) 8:531. 10.3389/fimmu.2017.00531 28572801 PMC5435759

