# Supplementary figures and images for: Computational Intelligence Technique for Prediction of Multiple Sclerosis Based on Serum Cytokines
Source: Front Neurol. 2019 Jul 18;10:781. doi: 10.3389/fneur.2019.00781 (PMC6657366; doi:10.3389/fneur.2019.00781)

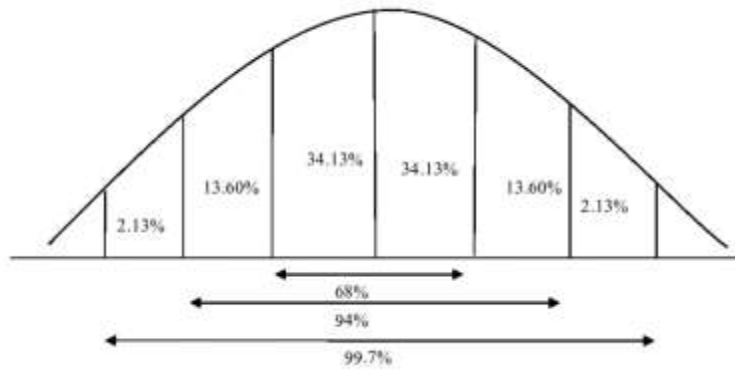

Supplementary figure 1: Standard normal distribution curve

Supplement: Supplementary file 1 [file Data_Sheet_1.PDF]
